# *Fasciola hepatica*: can the coproantigen ELISA replace the faecal egg sedimentation test?

**DOI:** 10.18849/ve.v9i4.698

**Published:** 2024-11-28

**Authors:** Jake Collyer

**Affiliations:** University of Surrey, United Kingdom

**Keywords:** cattle, coproantigen, cows, elisa, fasciola, fluke, sedimentation, sensitivity

## Abstract

**PICO question:**

In adult cattle, is the sensitivity of the coproantigen ELISA test equal or superior to the sensitivity of the faecal egg sedimentation test for the diagnosis of *Fasciola hepatica*?

**Clinical bottom line:**

**Category of research:**

Diagnosis.

**Number and type of study designs reviewed:**

Three studies were appraised. This included two cross-sectional diagnostic accuracy studies and one case control diagnostic accuracy study.

**Strength of evidence:**

Moderate.

**Outcomes reported:**

The first study reported the findings from 619 tested cattle over 3 sample periods comparing the sensitivity and specificity of the different tests. The sensitivity of the faecal egg sedimentation test varied greatly between the sample periods from 0.81 (95% beta coefficient (BCI) 0.72–0.90) to 0.58 (95% BCI 0.43–0.72) with the coproantigen ELISAs sensitivity remaining consistent at 0.77 (95% BCI 0.64–0.88) throughout.

The second study reported the findings of 200 tested cattle over 2 sampling periods comparing the sensitivity and specificity of the different tests. The mean sensitivity of the coproantigen ELISA was significantly higher than the 4 g/10 g preparations of the faecal egg sedimentation tests at 94% (95% CI 87%–98%) (P < 0.001).

The third study reported the findings of Coproantigen ELISA testing on 250 bovine faecal samples with 94 confirmed positive for liver fluke via faecal sedimentation testing. The sensitivity of the coproantigen ELISA was 80% and the specificity was 100% compared with 70% and 80% respectively for the faecal egg sedimentation test.

**Conclusion:**

All three studies demonstrated either an increased or equivalent sensitivity of the coproantigen ELISA to the faecal sedimentation test, but only one study reported a statistically significant increase in test sensitivity. Whilst all three studies were diagnostic accuracy validity studies, the systematic sampling strategy of one study was superior to the convenience sampling method of one of the other studies and to the case control method of the other.

Several sources of bias also exist within the included studies. Sampling and selection bias is present in the two of studies due to the animals selected only being sampled over one year. The results of these studies are susceptible to changes in the fluke lifecycle of that year, and the sampled animals are more likely to be fit and well-conditioned as they are presenting for slaughter, and as such are less likely to carry significant/chronic fluke burdens. All three studies are susceptible to validity issues due to an absence of clinical information regarding flukicide treatment and concurrent parasitic diseases which, whilst not impacting the efficacy of diagnostic testing, may cause issues if the studies are to be repeated.

The coproantigen ELISA can be utilised as a suitable adjunctive test to aid in the diagnosis of *Fasciola hepatica* in adult cattle and has the scope to be used as an early diagnostic test, but whilst the results of the reported studies indicate that the coproantigen ELISA is an accurate and reliable test, it does not provide definitive evidence to warrant the discontinuation of the simple and affordable faecal egg sedimentation test. In order to come to a conclusion regarding the more sensitive test more literature is required that directly compares the coproantigen ELISA to the faecal egg sedimentation test in different clinical scenarios and exploring different diagnostic techniques.

## How to apply this evidence in practice

The application of evidence into practice should take into account multiple factors, not limited to: individual clinical expertise, patient’s circumstances and owners’ values, country, location or clinic where you work, the individual case in front of you, the availability of therapies and resources.

Knowledge Summaries are a resource to help reinforce or inform decision-making. They do not override the responsibility or judgement of the practitioner to do what is best for the animal in their care.

## The evidence

A literature search found two cross-sectional diagnostic accuracy studies (Charlier et al., (2008) and Mazeri et al., (2016)) and one case control diagnostic accuracy study (Palmer et al., 2014). These studies evaluated the diagnostic accuracy and therefore sensitivity and specificity of the different diagnostic tests with the focus in this Knowledge Summary being the comparison of the sensitivity of the faecal sedimentation test (FEST) to the coproantigen enzyme-linked immunosorbent assay (ELISA).

Mazeri et al. (2016) utilised a systematic sampling strategy to select study participants, Charlier et al. (2008) utilised a convenience sampling strategy to select its study participants, and Palmer et al. (2014) utilised a case control sampling strategy.

Charlier et al. (2008) and Mazeri et al. (2016) focused on comparing the accuracy of multiple diagnostic tests with Mazeri et al. 2016 utilising a Bayesian no universally accepted standard approach for test comparison, and Charlier et al. (2008) using liver necropsy as its standard. Palmer et al. 2014 compared the accuracy of the coproantigen ELISA tests against identification of liver fluke eggs on faecal sedimentation as its standard. The strength of evidence of the included papers is moderate as described in the following sections.

## Summary of the evidence

**Charlier et al. (2008):** 

Population	Cattle of mixed beef and dairy breeds, designated for slaughter at Brugge abattoir, Belgium, were recruited over 2 sampling periods (Feb-May 2006, and Oct-Dec 2006). Cows ≥ 24 months of age. February-May (Spring) 2006 – 100 cattle. October-December (Autumn) 2006 – 100 cattle.
Sample Size	200 cattle over 2 sampling periods.
Intervention Details	No more than 3 cattle were selected from the same farm. Faeces, blood, whole livers, and gall bladders were obtained following slaughter from each sampled animal. Whole livers were individually visually assessed, dissected, and allocated fibrosis scores ranging from none to severe (0–3); flukes were collected if present. Two serum *Fasciola hepatica* antibody enzyme-linked immunosorbent assay (ELISAs) were performed on collected blood samples following manufacturer specifications: An in-house ELISA. A commercial ELISA (Institut Pourquier, France). A γ-glutamyl transferase (GGT) measurement was also performed. Two faecal egg sedimentation tests were performed on each faecal sample to identify *F. hepatica* eggs: 4 g preparation. 10 g preparation. A coproantigen ELISA was performed on each faecal sample using the *F. hepatica *antigen ELISA kit (Bio-X Diagnostics, Belgium) according to manufacturer specifications and cut-offs. Statistical analysis was performed to calculate individual test sensitivity and specificity compared with fluke counts on liver necropsy as standard.
Study design	Cross-sectional diagnostic accuracy study.
Outcome studied	Objective assessment: Positive and negative results of each diagnostic test for the presence of *F. hepatica* in each sample period. Cattle were deemed positive on identification of ≥ 1 fluke egg identified in the faecal sedimentation, ≥ 1 fluke identified in the liver or according to test cut-off values for serological antibody/coproantigen ELISA. Prevalence of positive liver fluke cases in each sample population. Characterisation of fluke burden based on necropsy. Calculated sensitivity and specificity of each diagnostic test when compared with liver necropsy as standard. Autumn sensitivity and specificity. Spring sensitivity and specificity. Mean sensitivity and specificity. Calculated likelihood ratios for each diagnostic test. Likelihood ratio for positive test. Likelihood ratio for negative test.
Main findings (relevant to PICO question)	The mean sensitivity of the coproantigen ELISA was more sensitive than that of the two faecal egg sedimentation tests: Coproantigen – 94% (95% CI 87%–98%) 4 g Faecal egg sedimentation– 43% (95% CI 33%–54%) 10 g Faecal egg sedimentation – 64% (95% CI 53%–74%). The coproantigen ELISA test sensitivity was significantly different from either of the faecal egg sedimentation tests (P < 0.001). The sensitivity of the 10 g preparation for the faecal egg sedimentation test was significantly different from the 4 g preparation (P < 0.005).
Limitations	False positive results possible from other parasitic infections. Unknown flukicide treatment history. Convenience sampling resulting in unrepresentative sample populations. Sampled cattle may not be representative as unlikely to have significant burdens if healthy and presenting for slaughter to enter the food chain. Sampled cattle may vary in geographic location, management practices, and grazing strategies. Small sample size.

Table note...

**Mazeri et al. (2016):** 

Population	Cattle designated for slaughter at Scotbeef Limited abattoir. Cattle were sourced from farms in Scotland, Northern Ireland, and the North of England over 3 sampling periods: A, B & C Summer (June–July) 2013 – 207 cattle. Winter (January–March) 2014 – 204 cattle. Autumn (August–October) 2014 – 208 cattle. Cattle ages were 369–1121 days. Cattle were of mixed breeds. Systematic sampling was conducted with 1 in every 10 cattle being selected for inclusion into the study.
Sample Size	619 cattle over 3 sampling periods.
Intervention Details	Sampling conducted over 1 day a week for 6 weeks in each sampling period. Blood, faeces, whole livers, and gall bladders were obtained following slaughter from each sampled animal. Whole livers were assessed for signs of fluke by the meat hygiene service via visual assessment and palpation. Whole livers were dissected, and fibrosis scores allocated to each, from none to severe (0–3), alongside whole flukes being collected from the cut surfaces. Gall bladder egg counts were performed via sedimentation and microscopic analysis of content. Serum *F. hepatica* antibody enzyme linked immunosorbent assays (ELISAs) were performed according to developer specifications with two modifications: 1:8000 monoclonal mouse anti-bovine Immunoglobulin G (IgG) conjugate was used. A different positive control was used in the results equation. Faecal egg sedimentation tests were performed on 5 g of collected faeces via the faecal egg sedimentation technique. Coproantigen ELISAs were performed on faeces using the *F. hepatica* antigen ELISA kit (Bio-X Diagnostics, Belgium) according to manufacturer specifications and cut-offs. Statistical analysis was performed via a Bayesian no standard approach to determine individual test sensitivity and specificity.
Study design	Cross-sectional diagnostic accuracy study.
Outcome studied	Objective assessment: Positive and negative results of each diagnostic test for the presence of *F. hepatica* in each sampling period. Cattle were deemed positive on identification of ≥ 1 fluke egg identified in the faecal sedimentation/gallbladder sedimentation, ≥ 1 fluke identified in the liver, or according to test cut-off values for serological antibody/coproantigen ELISA. Individual test determined prevalence of *F. hepatica* within the sample populations within each sampling period. Individual fluke burden assessment based on liver necropsy. An estimation of individual test sensitivity and specificity in each sampling period utilising Bayesian methods without a standard test to allow for comparison.
Main findings (relevant to PICO question)	The coproantigen ELISA sensitivity remained constant between the three sample periods: Summer – 0.77 (95% beta coefficient (BCI) 0.67–86). Winter – 0.77 (95% BCI 0.67–87). Autumn – 0.77 (95% BCI 0.64–88). The faecal egg sedimentation tests sensitivity varied between the three sample periods: Summer – 0.81 (95% BCI 0.72–90). Winter – 0.77 (95% BCI 0.66–86). Autumn – 0.58 (95% BCI 0.43–0.72).
Limitations	Systematic sampling susceptible to bias. Sampling limited to one year – does not address variances in liver fluke cycle due to climatic factors or variances in fluke cycle. Sampled cattle may not be representative as unlikely to have significant burdens if healthy and presenting for slaughter to enter the food chain. Sampled cattle may vary in geographic location, management practices and, grazing strategies. Validity Issues: Unknown flukicide treatment history. False positive results possible from concurrent parasitic infections.

Table note...

**Palmer et al. (2014):** 

Population	Faecal samples collected from cattle, sheep and equids: Faecal samples negative for *F. hepatica* were sourced from animals in Western Australia (WA) where *F. hepatica* is not present. Positive faecal samples were sourced from animals testing positive via faecal sedimentation testing from laboratories outside of WA or from animals testing positive prior to entry into the state. Samples were collected and tested between 2008–2012.
Sample Size	250 cattle faecal samples: 156 negative 94 positive. 146 sheep faecal samples: 106 negative 40 positive. 176 equine faecal samples: 110 negative 67 positive. Results for the sheep and equine samples will not be reported as the results are not relevant to the PICO question.
Intervention Details	All sampled faeces were subjected to coproantigen. Samples were prepared according to *F. hepatica* antigen ELISA (enzyme-linked immunosorbent assay) kit protocol (Bio K 201, Bio-X Diagnostics, Belgium) with some modifications: Samples were vortexed after the addition of the dilution buffer. Samples were extracted overnight at 4–8°C. Samples were centrifuged and supernatant was collected. Test sensitivities were calculated from the known positive and negative faecal samples. Negative samples were obtained from animals in geographical locations where* F. hepatica *does not occur.
Study design	Case control diagnostic accuracy study.
Outcome studied	Objective assessment: Positive and negative results for coproantigen ELISA testing based on recommended cut-off values in coproantigen ELISA test kits and custom lowered test cut-off values. Calculated sensitivity of the coproantigen ELISA test kits against the results of the faecal sedimentation as a standard when the manufacturer test cut-off was applied for cattle. Estimated sensitivity of the coproantigen ELISA test kits against a standard when a custom lowered test cut-off was applied for cattle.
Main findings (relevant to PICO question)	The bovine coproantigen ELISA test sensitivity was high (80%) but had a 20% false negative rate when recommended test cut-off values were applied.
Limitations	Case control sampling methods do not provide a strong basis of evidence. Test kit protocol was changed midway through study. Confidence interval and significance were not reported in study. It was not specified if negative controls are confirmed with FEST testing. No comparison with the faecal egg sedimentation test – only an estimation of coproantigen ELISA sensitivity based on utilising the faecal sedimentation test as a standard. Flukicide treatment history not specified. Age/season/grazing status not specified.

Table note...

## Appraisal, application and reflection

Liver fluke (*F. hepatica*) is one of the most important endoparasites affecting livestock within the UK, associated with reduced productivity, increased time to slaughter, liver rejections, and occasional sudden death within herds (Cawdery et al., 1977; Mazeri et al., 2017). The principal concern comes from the apparent increase in cases in recent years as shifting weather patterns lead to alterations in the fluke lifecycle, resulting in unpredictable risk-periods for infection (Skuce and Zadoks, 2013). Therefore, it is more important than ever to have an accurate and reliable test that can provide a definitive diagnosis in order to allocate flukicide treatment/preventative measures appropriately. An evidence review was warranted as no diagnostic test for *F. hepatica* has been recorded as having 100% sensitivity and specificity (Rapsch et al., 2006), it is important, therefore, to be able to evaluate the accuracy and reliability of the diagnostic tests available in order to advise on which test/combination of tests should be prioritised to obtain a valid diagnosis.

The faecal egg sedimentation test has been a longstanding, affordable and effective test for the detection of *F. hepatica* for many years. Demonstrating a near perfect test specificity in many studies (Graham-Brown et al., 2019; Reigate et al., 2021), it has historically been the universally accepted test in the diagnosis of liver fluke in farm animal species; however, the faecal egg sedimentation test falls short when considering the test sensitivity as various studies have reported wildly different sensitivities indicating that a negative test result is not definite for being free from infection (Anderson et al., 1999; Arifin et al., 2016; Graham-Brown et al., 2019). Various reasons for this relative insensitivity exist, the foremost of these being the variability with shedding of the *Fasciola* eggs into the biliary system, which is dependent on the presence of mature flukes within the liver, which itself is dependent on the environmental conditions such as temperature, humidity, and rainfall to propagate the snail intermediate host, and the burdens of *Fasciola* in affected cattle that influence the number of eggs being shed in faeces at any one time (Beesley et al., 2018; Charlier et al., 2014).

The coproantigen enzyme-linked immunosorbent assay (ELISA) test is another diagnostic test that can be performed on samples of faeces from cattle, with the Bio X Diagnostics (Belgium) *F. hepatica* test kit being one commonly utilised test. Likewise, multiple studies have reported near perfect test specificity (Kajugu et al., 2012; Kajugu et al., 2015), but data regarding test sensitivity is more variable, similar to the faecal egg sedimentation test. Theoretically the test sensitivity should not be affected by the life stage of the infecting liver fluke or the variability in egg shedding in the faeces, so should be more useful in the detection of early *F. hepatica* infection (Mezo et al., 2004). This testing methodology does come with several disadvantages, including higher running costs when compared to the faecal egg sedimentation test, a requirement for more advanced laboratory equipment, and diagnostic cut-off values set by the test manufacturer.

This Knowledge Summary compared the sensitivities of the above-mentioned diagnostic techniques to determine if the commonly used faecal egg sedimentation test (FEST) is still an appropriate standard test to perform given the range of other diagnostic tests available. Three papers were identified for inclusion into this Knowledge Summary that addressed the PICO question, two of which reported the results from multiple diagnostic methods on animals designated for slaughter at different slaughterhouses, with the third (Palmer et al., 2014) reporting the findings of coproantigen ELISA testing on known positive faecal egg samples when compared to faecal samples obtained from *F. hepatica*-free areas.

Whilst all studies reported a coproantigen ELISA test specificity equal to or greater than that of the faecal egg sedimentation test, the sensitivity was significantly increased in only one study (Charlier et al., 2008). Mazeri et al. (2016) exhibited an increased sensitivity in one out of three sampling periods and an equivalent sensitivity in another period, and Palmer et al. (2014) demonstrated an increase in test sensitivity of 80% compared to the reference value of 70%.

Charlier et al. (2008) conducted a convenience sampling methodology, sampling the first 10–12 cattle that met the inclusion criteria and allowing for up to 3 cattle from the same herd to be sampled. A relatively small sample size was selected for each sampling period, potentially reducing the power of the study and opening the study to error. Similar to the previous study, inclusion criteria were outlined for the sampled cattle and sampling was conducted over multiple periods, allowing for seasonal variation to be accounted for but as this period is only limited to one year it does not account for variations in the lifecycle of *F. hepatica*.

The sample populations for Mazeri et al. (2016) and Charlier et al. (2008) are conducted using clinically healthy cattle presented for slaughter. These cattle are therefore likely to be well conditioned and unlikely to be carrying significant/chronic fluke burdens. As such they may not be representative of the cattle that would be tested in clinical scenarios, therefore sensitivity of the faecal sedimentation test is likely to be low when compared to the sensitivity of the coproantigen ELISA test as it has been shown to be accurate down to burden of only one fluke.

Palmer et al. (2014) provides a weak body of evidence for this Knowledge Summary and does not entirely address the PICO question. The quality of evidence supplied is reduced by; the case control sampling method, a relatively small positive sample size, failure to include relevant inclusion criteria of submitted faecal samples such as age/grazing status, failure to include confidence intervals when reporting test accuracy, and the modification of the test protocol midway through the study.

Palmer et al. (2014), Mazeri et al. (2016), and Charlier et al. (2008) are open to several sources of validity issues. Namely, they carried an unknown history of flukicide treatment at the time of sampling, potentially affecting the results of each diagnostic test, and an unknown parasite status at the time of sampling. Additionally, geographical location, management, and grazing strategies are not specified in the inclusion criteria of any of the three studies.

All three studies reported thorough faecal collection, storage, and test protocols, including relevant cut-off values for positive results. Additionally, all studies were performed with populations naturally infected with *F. hepatica* and were therefore more akin to findings identified in clinical practice.

In conclusion, the evidence reviewed provides a moderate argument for utilising the coproantigen ELISA but is not sufficient to be able to recommend a total replacement of the faecal egg sedimentation test for the diagnosis of *F. hepatica* in cattle just yet, due to the relatively insufficient body of evidence available and limitations of the studies as discussed. This is an economically important disease within the livestock industry and as such the basis of evidence requires significant further development and expansion before any clear conclusions can be drawn due to multiple confounding factors that influence the results of the diagnostic tests. It is; however, important to note the significant role the coproantigen ELISA has to play as an adjunctive test when diagnosing pre-patent infections with *F. hepatica*, and additional testing should always be considered when faced with cases of high suspicion testing negative.

## Methodology

**Search Strategy:** 

Databases searched and dates covered	CAB Abstracts on OVID from 2000 May 2024 Scopus on OVID from 2000 May 2024
Search terms	CAB Abstracts: Search Field: Abstract (Fasciola-hepatica OR liver-fluke OR fasciolosis) (Bovine OR bovid OR cattle OR cow OR cows) (Coproantigen-ELISA OR coproantigen OR copro-antigen OR copro-antigen-ELISA) (Faecal-egg OR fecal-egg OR FEC OR sedimentation) 1 AND 2 AND 3 AND 4 Scopus: ABS ( ( fasciola-hepatica OR liver-fluke OR fasciolosis ) AND ( bovine OR bovid OR cattle OR cow OR cows ) AND ( coproantigen-elisa OR coproantigen OR copro-antigen OR copro-antigen-elisa ) AND ( faecal-egg OR fecal-egg OR fec OR sedimentation) )
Dates searches performed:	25 May 2024

Table note placeholder

**Exclusion / inclusion criteria:** 

Exclusion	Unavailable in English language. Findings not relevant to PICO question. Unable to access full text. Non-peer reviewed articles, book chapters, reports, conference proceedings.
Inclusion	Comparative studies of diagnostic testing. Reported sensitivities of the diagnostic tests.

Table note placeholder

**Search Outcome:** 

Database	Number of results	Excluded – unavailable in English language	Excluded – does not address the PICO question	Excluded– unable toaccess full text	Total relevant papers
CAB Abstracts	26	4	16	3	3
Scopus	21	3	13	2	3
Total relevant papers when duplicates removed					3

Table note placeholder

## ORCiD

Jake Collyer: https://orcid.org/0009-0008-8147-7789

## Conflict of interest

The author declares no conflicts of interest.

## Disclaimer

Knowledge Summaries are a peer-reviewed article type which aims to answer a clinical question based on the best available current evidence. It does not override the responsibility of the practitioner. Informed decisions should be made by considering such factors as individual clinical expertise and judgement along with patient’s circumstances and owners’ values. Knowledge Summaries are a resource to help inform and any opinions expressed within the Knowledge Summaries are the author’s own and do not necessarily reflect the view of the RCVS Knowledge. Authors are responsible for the accuracy of the content. While the Editor and Publisher believe that all content herein are in accord with current recommendations and practice at the time of publication, they accept no legal responsibility for any errors or omissions, and make no warranty, express or implied, with respect to material contained within. For further information please refer to our Terms of Use.
